# Effects of time-restricted eating without caloric restriction on blood pressure and cardiometabolic profile in non-diabetic adults: a systematic review and meta-analysis of randomized controlled trials

**DOI:** 10.3389/fnut.2025.1631477

**Published:** 2025-11-19

**Authors:** Xin Yi, Jie Yan, Ummi Nadira Daut, Razif Abas, Raja Abdul Wafy Raja Muhammad Rooshdi, Chongshuang Yang, Canzhang Liu

**Affiliations:** 1 Department 1 of Cardiovasology, North China University of Science and Technology Affiliated Hospital, Tangshan, Hebei, China; 2 Department of Internal Medicine, Faculty of Medicine and Health Sciences, Universiti Putra Malaysia, Serdang, Selangor, Malaysia; 3 Department of Human Anatomy, Faculty of Medicine and Health Sciences, Universiti Putra Malaysia, Serdang, Selangor, Malaysia; 4 Department of Radiology, Tongren People's Hospital, Tongren, Guizhou, China

**Keywords:** time-restricted eating, Cardiometabolic risk, blood pressure, insulin resistance, systematic review

## Abstract

**Objective:**

To systematically evaluate the effect of time-restricted eating (TRE) without caloric restriction (CR) on blood pressure (BP) and cardiometabolic profile in non-diabetic adults.

**Methods:**

A comprehensive search of electronic databases identified 978 potentially relevant studies, of which 11 randomized controlled trials involving 653 participants were included in this meta-analysis. All included studies compared time-restricted eating (TRE; 6–10-h daily eating windows) without CR to unrestricted feeding controls. Outcomes assessed included systolic BP (SBP), diastolic BP (DBP), heart rate (HR), fasting blood glucose (FBG), fasting insulin (FINS), homeostasis model assessment of insulin resistance (HOMA-IR), and body mass index (BMI). Weighted mean differences (WMD) and 95% confidence intervals (CI) were calculated using random-effects models.

**Results:**

TRE without CR significantly reduced SBP (WMD = −1.79 mmHg, 95% CI: −3.30 to −0.27, *p* = 0.02), DBP (WMD = −1.75 mmHg, 95% CI: −3.07 to −0.43, *p* = 0.01), and HR (WMD = −2.19 bpm, 95% CI: −4.01 to −0.36, *p* = 0.02). Subgroup analyses showed greater BP reductions in participants with elevated baseline SBP or DBP. TRE also led to significant improvements in metabolic parameters, including reductions in FBG (WMD = −2.65 mg/dL, 95% CI: −3.92 to −1.39, *p* < 0.0001), FINS (WMD = −2.00 μIU/mL, 95% CI: −3.02 to −0.97, *p* = 0.0001), HOMA-IR (WMD = −0.58, 95% CI: −0.81 to −0.35, *p* < 0.00001), and BMI (WMD = −1.59 kg/m^2^, 95% CI: −2.98 to −0.20, *p* = 0.03). Heterogeneity across outcomes was negligible to moderate.

**Conclusion:**

TRE without CR can significantly reduce BP and improve glucose metabolism in non-diabetic adults, particularly in those with pre-existing high BP or high FBG or High FINS. However, TRE does not appear to exert meaningful effects on lipid profiles in the absence of CR, indicating that its cardiometabolic benefits may be selective rather than comprehensive. These findings support the potential of TRE as a non-pharmacological intervention for cardiometabolic health, particularly in populations with elevated baseline risk markers.

**Systematic review registration:**

https://www.crd.york.ac.uk/PROSPERO, identifier CRD420251052403.

## 1 Introduction

Cardiovascular diseases (CVDs) persist as a leading cause of global disease burden, contributing to elevated rates of disability-adjusted life years (DALYs) across populations. Recent epidemiological data indicate that CVDs-related fatalities exceed 17 million annually, constituting nearly one-third of total global mortality (1). Hypertension and cardiometabolic dysfunction are widely recognized as key modifiable drivers of CVDs pathogenesis, as they can lead to endothelial dysfunction, vascular stiffness, and inflammatory responses, all of which contribute to the development of atherosclerosis, heart failure, and stroke (2–4), underscoring the importance of their management in primary and secondary prevention (5).

Caloric restriction (CR), a dietary strategy involving reduced energy intake while maintaining nutritional adequacy, has demonstrated efficacy in modulating cardiovascular risk markers. Clinical evidence confirms its ability to lower blood pressure (BP), improve glycemic control, and optimize lipid metabolism (6, 7). Nevertheless, CR’s utility in real-world settings is constrained by the necessity for precise caloric tracking and sustained behavioral modification, factors frequently associated with suboptimal long-term adherence (8).

Time-restricted eating (TRE), a variant of intermittent fasting, has emerged as a patient-friendly alternative by limiting food consumption to a defined daily window (typically 6–12 h) without mandatory CR (9, 10). By aligning eating patterns with circadian rhythms and enhancing metabolic efficiency, TRE may exert cardiovascular benefits through the reduction of oxidative stress and vascular inflammation. It may also enhance compliance by preserving meal flexibility while still conferring metabolic advantages (11). Early research reports TRE-associated improvements in BP regulation, insulin sensitivity, and dyslipidemia (12–14), though findings remain inconsistent.

Current controversies in the field highlight divergent outcomes, with some trials documenting significant BP reductions (12, 14) while others report null effects (15, 16). A critical methodological concern involves the frequent conflation of TRE with concurrent CR, obscuring the independent effects of temporal feeding patterns. Diabetes mellitus further complicates this relationship, as disease management often necessitates strict energy intake control, potentially masking TRE-specific impacts.

To resolve these uncertainties, we propose a systematic evaluation of RCTs examining TRE’s isolated effects on cardiometabolic parameters in non-diabetic cohorts, explicitly excluding studies incorporating CR. This meta-analytic approach will clarify whether observed benefits stem from feeding timing per se rather than concomitant energy reduction. By addressing CR’s practical limitations through investigation of a more sustainable intervention, our findings may inform novel dietary strategies for CVDs risk mitigation.

## 2 Materials and methods

The methodology of this review adhered to the Preferred Reporting Items for Systematic Reviews and Meta-Analyses (PRISMA) statement (17). The review protocol was registered with the International Prospective Register of Systematic Reviews (PROSPERO) to ensure transparency and methodological rigor. Record identification: CRD420251052403.

### 2.1 Search strategy

We conducted systematic searches in PubMed, Embase, Cochrane Library, and Scopus (up to April 27, 2025) using the following combined terms: (“time-restricted eating” OR “Time restricted feeding”) AND (“blood pressure” OR “systolic blood pressure” OR “diastolic blood pressure” OR “cholesterol” OR “triglycerides” OR “LDL” OR “HDL” OR “total cholesterol” OR “blood glucose” OR “HbA1c” OR “insulin resistance” OR “metabolic syndrome” OR “cardiometabolic risk factors” OR “body composition” OR “body weight” OR “weight loss” OR “body mass index” OR “fat mass” OR “waist circumference”) AND “randomized controlled trial.” All search results were exported to EndNote 21 (Clarivate Analytics) for duplicate removal. Following deduplication, two independent reviewers (Xin Yi and Jie Yan) screened the records by title/abstract and subsequently by full-text against the eligibility criteria. Consensus was reached through discussion at each screening stage, with any remaining disagreements resolved by a third reviewer (Canzhang Liu). To ensure comprehensive coverage, we supplemented database searches by manually screening reference lists of included studies.

### 2.2 Study selection

#### 2.2.1 Inclusion criteria

The systematic review incorporated studies meeting the following requirements: (1) Only randomized controlled trials (RCTs) were included to ensure high-quality evidence; (2) Participants were restricted to adults (≥18 years) without diabetes mellitus, as diabetic populations require specific dietary management that could confound TRE effects; (3) The intervention group implemented TRE with a clearly defined eating window ≤12 h/day without CR for ≥4 weeks duration; (4) Control groups maintained normal unrestricted eating patterns (≥12-h window) without any dietary limitations. (5) If multiple TRE intervention arms were present, we prioritized the group with the longest eating window. When multiple timing schedules existed, we selected either self-selected timing or the protocol closest to typical human eating patterns, as this approach minimizes fasting duration and may enhance patient adherence.

#### 2.2.2 Exclusion criteria

Studies were excluded based on these considerations: (1) Any study involving diabetic populations or other conditions requiring specialized dietary management; (2) Interventions combining TRE with exercise programs, special diets, or other lifestyle modifications; (3) Studies implementing any form of CR in either group; (4) Non-randomized study designs, including observational studies or uncontrolled trials; (5) Non-English publications.

#### 2.2.3 Selection process

A systematic screening process was conducted in duplicate by two independent reviewers (Xin Yi and Jie Yan). In the first phase, all identified records were screened based on titles and abstracts using the predefined eligibility criteria. In the second phase, full-text articles of potentially relevant studies were thoroughly assessed against the inclusion/exclusion criteria. Any disagreements between reviewers were resolved through discussion or, when necessary, by consultation with a third senior investigator (Canzhang Liu). The entire selection process was carefully documented using a PRISMA flow diagram, with specific reasons for exclusion recorded at each screening stage.

Key outcome measures of interest included: BP parameters, including systolic BP (SBP) and diastolic BP (DBP), heart rate (HR), body mass index (BMI), and comprehensive metabolic markers, including fasting blood glucose (FBG), fasting insulin (FINS), homeostasis model assessment of insulin resistance (HOMA-IR), and complete lipid profiles including total cholesterol (TC), triglycerides (TG), LDL-cholesterol (LDL-C), and HDL-cholesterol (HDL-C). The minimum 4-week intervention duration requirement ensured sufficient time for metabolic adaptations to be observed while maintaining clinical relevance for real-world application.

### 2.3 Risk of bias (ROB) assessment

The methodological quality of included RCTs was evaluated by two independent reviewers (Xin Yi and Jie Yan) using the Cochrane ROB tool (RoB 2.0), assessing five key domains: (1) randomization process, (2) deviations from intended interventions, (3) missing outcome data, (4) outcome measurement, and (5) selective reporting. Each study was classified as “low risk,” “some concerns,” or “high risk” following the official RoB 2.0 algorithms. Any disagreements between reviewers were resolved through discussion or, when necessary, by consultation with a third senior investigator (Canzhang Liu). Where methodological details were unclear, corresponding authors were contacted for clarification. Final judgments incorporated consideration of trial registration documents and protocols when available, and overall study-level risk was determined by the most critical rating across domains. All assessments were visualized using standardized risk-of-bias plots generated with the Robvis package.

### 2.4 Data extraction

Two independent reviewers (Xin Yi and Jie Yan) extracted data using a standardized form. Any disagreements between reviewers were resolved through discussion or, when necessary, by consultation with a third senior investigator (Canzhang Liu). We collected: (1) study characteristics (author, year, design, sample size, country); (2) participant demographics (age, gender, baseline parameters); (3) intervention details (TRE/control protocols, eating windows, duration); and (4) outcome measures (BP, lipids profile, glucose metabolism). For studies reporting means with 95% CIs, we converted to SDs using Cochrane Handbook formulae. For non-parametric data (medians/IQRs), values were converted to mean±SD using established methods (18). Missing SDs were estimated from SEs/CIs when necessary. All conversions were independently verified.

### 2.5 Meta-analysis

We analyzed continuous outcomes using weighted mean difference (WMD) with 95% confidence intervals (CIs) based on outcome measurement consistency across studies, employing random-effects models as the primary approach to account for anticipated clinical and methodological heterogeneity in TRE protocols (particularly variations in feeding windows and intervention durations). Meta-analyses were performed when ≥3 studies reported comparable outcomes, with results presented in forest plots (statistical significance threshold *p* < 0.05). Heterogeneity was assessed using I^2^ statistics (I^2^ > 60% considered substantial), which prompted pre-specified sensitivity analyses including: (1) sequential exclusion of individual studies to evaluate their influence on pooled effects, (2) exclusion of studies involving populations with extreme baseline metabolic characteristics, and (3) restriction to studies with low risk of bias. Publication bias was evaluated through funnel plot asymmetry for outcomes with ≥10 studies. All analyses were conducted using RevMan 5.4 (Cochrane Collaboration).

## 3 Results

### 3.1 Literature screening

**Figure 1:**
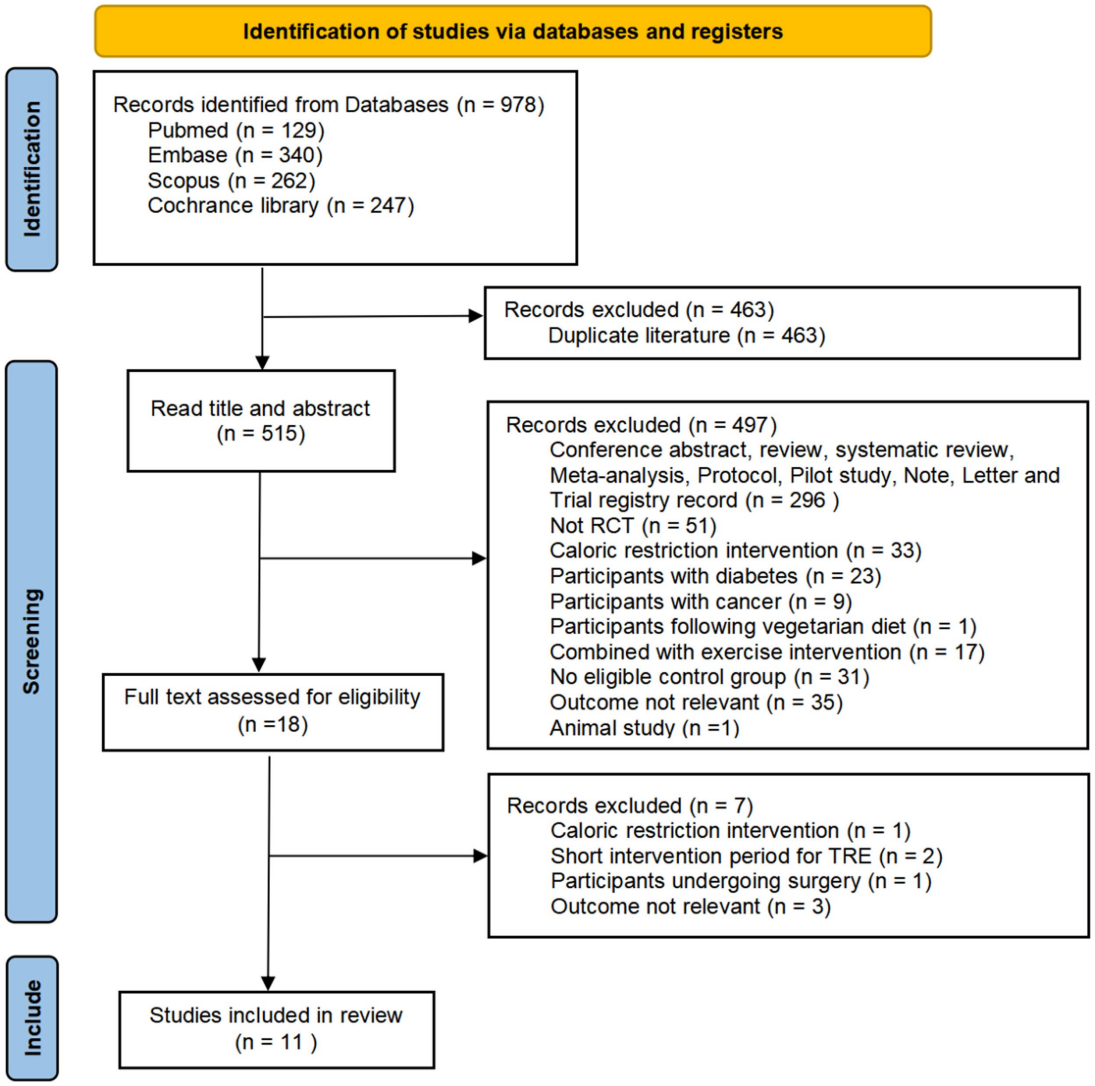
PRISMA flow diagram.

Our systematic search across electronic databases initially yielded 978 potentially relevant citations. After removing 463 duplicate records, we screened the remaining 515 unique studies based on titles and abstracts, which led to the exclusion of 497 ineligible studies. Following detailed evaluation of the full texts of 18 potentially eligible articles, we excluded 7 additional studies that did not meet our predefined inclusion criteria. Ultimately, 11 studies qualified for inclusion in our meta-analysis. The complete study selection procedure is presented in Figure 1.

### 3.2 Basic features of studies

The baseline characteristics of participants across the included studies are shown in Table 1. This meta-analysis incorporated 653 adult participants with overweight/obesity, stage-1 hypertension, metabolic syndrome, or impaired fasting glucose. All studies included at least one TRE intervention group compared with an unrestricted feeding control group, with CR prohibited in both groups. In all studies, as no participants in either the TRE or the control groups were instructed to alter their dietary composition, the dietary macronutrient pattern was both identical between groups and unchanged during the TRE intervention. The intervention duration ranged from 6 weeks to 12 months, with eating windows of 6–10 h. Feeding schedules were either participant-selected or investigator-defined, with all prescribed eating windows occurring between 08:00 and 20:00. Geographically, the majority of studies were conducted in the United States (*n* = 8), followed by China (*n* = 2) and Norway (*n* = 1).

**Table 1:** The basic features of studies.

Author (Year)	Sample size		Gender (F/M)		Age		Country	Population	TRE features				Dietary macronutrient pattern	
	CTR	TRE	CTR	TRE	CTR	TRE			Duration	Eating window	Timing	Meals / Day	CTR vs. TRE	During TRF
Zhou (2024) (27)	37	37	NR		49.194 (7.123)	47.514 (7.784)	China	Hypertension stage1	6 weeks	8 h	9:00–17:00	Unrestricted	Identical	Unchanged
Cienfuegos (2020) (26)	14	19	12/2	18/1	45 (2)	47 (3)	United States	Obesity	8 weeks	6 h	13.00–19.00	Unrestricted	Identical	Unchanged
Oldenburg (2025) (25)	29	30	16/13	17/13	43.4 (10.7)	44.0 (11.5)	United States	Obesity	12 weeks	10 h	Self-selected	Unrestricted	Identical	Unchanged
Chow (2020) (29)	9	11	1/8	2/9	44.2 (12.3)	46.5 (12.4)	United States	Overweight	12 weeks	8 h	Self-selected	Unrestricted	Identical	Unchanged
Haganes (2022) (24)	33	33	33/0	33/0	36.4 (6.2)	36.2 (5.9)	Norway	Women with overweight obesity	7 weeks	10 h	Self-selected	Unrestricted	Identical	Unchanged
Lowe (2020) (23)	57	59	22/35	24/35	46.1 (10.3)	46.8 (10.8)	United States	Overweight / obesity	12 weeks	8 h	12:00–20:00	Unrestricted	Identical	Unchanged
Cui (2025) (28)	12	15	10/2	9/6	20 (1)	20 (1.1)	China	College students with overweight / obesity	8 weeks	10 h	Self-selected	Unrestricted	Identical	Unchanged
Suthutvoravut (2023) (22)	22	24	16/6	16/8	55.2 (7.9)	55.5 (7.2)	United States	Patients with impaired fasting glucose	12 weeks	9 h	8:00–17:00	Unrestricted	Identical	Unchanged
Lin (2023) (21)	30	30	25/5	25/5	44 (13)	44 (12)	United States	Obesity	12 months	8/10 h	First 6 months: 12:00–20:00 Last 6 months: 10:00–20:00	Unrestricted	Identical	Unchanged
Manoogian (2024) (20)	61	61	31/30	31/30	60.6 (10.3)	56.6 (11.5)	United States	Metabolic syndrome	12 weeks	8–10 h	Self-selected	Unrestricted	Identical	Unchanged
Dote-Montero (2025) (19)	49	47	24/25	25/22	46.7 (6.0)	45.2 (5.8)	United States	Overweight / obesity	12 weeks	8 h	Self-selected	Unrestricted	Identical	Unchanged

CTR, Control group; TRE, Time-restricted eating group.

### 3.3 ROB assessment

**Figure 2:**
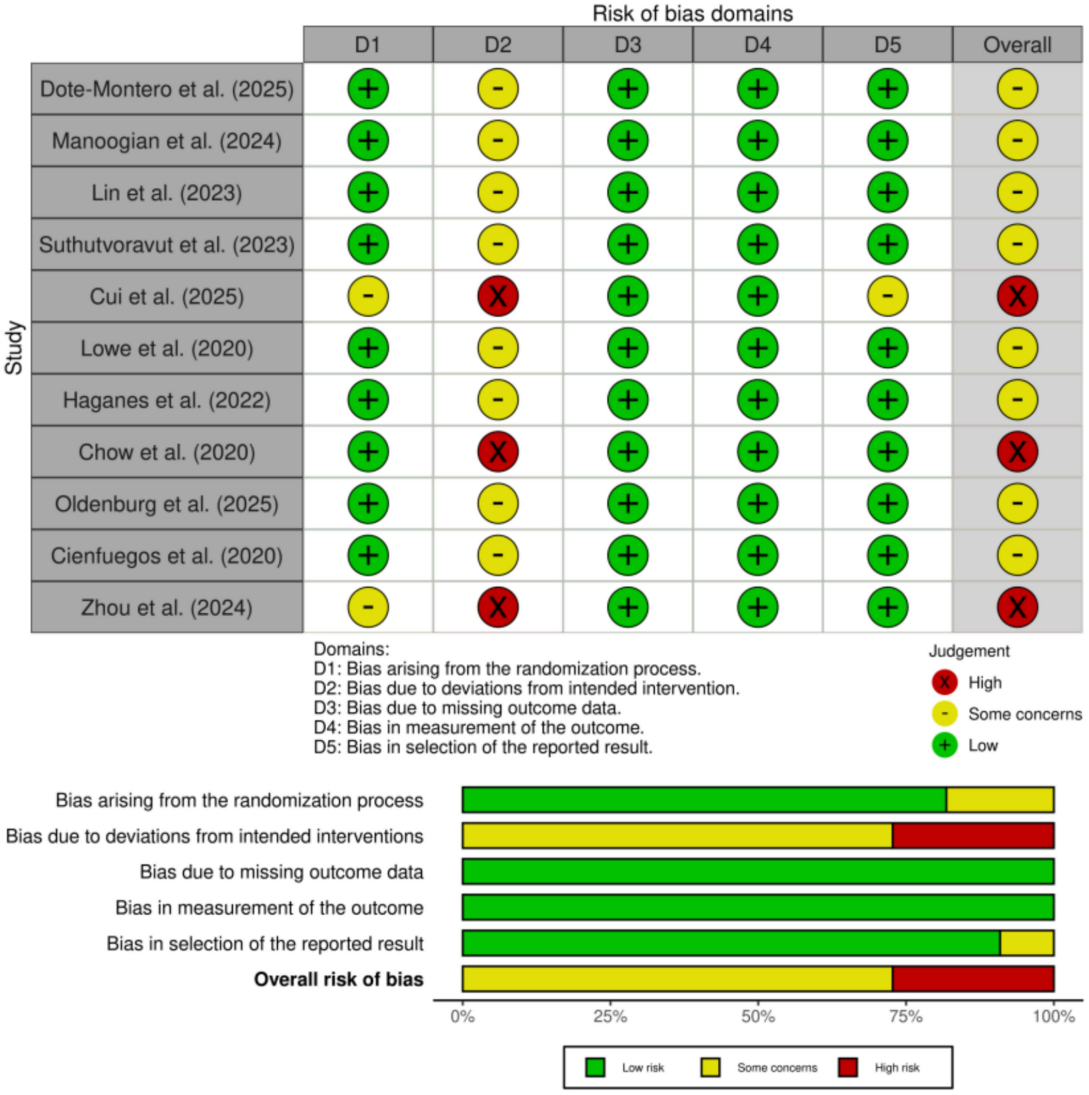
ROB assessment of the studies included in the meta-analyses.

Among the 11 included studies, 8 studies (19–27) were judged to have some concerns, and 3 studies (27–29) were assessed as having a high risk of bias overall. The most frequent source of bias was related to deviations from intended interventions (Domain 2), which is a common challenge in behavioral and dietary interventions where participant blinding is not feasible. This high-risk rating was attributable to participant non-adherence to the prescribed TRE regimen, which reflects a real-world implementation challenge rather than a deviation in the study protocol by the investigators. Bias due to the randomization process (Domain 1) was also identified in several studies (27, 28), primarily due to insufficient reporting of allocation concealment. All studies were rated as low risk in Domain 3 (bias due to missing outcome data), indicating nearly complete data collection and appropriate handling of attrition. Similarly, bias in measurement of outcomes (Domain 4) was consistently low, as most outcomes were objective and assessed using standardized methods. Selective reporting bias (Domain 5) was also judged to be low in the majority of studies (28), with only isolated concerns. Overall, the methodological quality of the included studies was considered acceptable. Although some studies exhibited domain-specific risks of bias, these are unlikely to have materially affected the reliability of the pooled estimates in this meta-analysis. As shown in Figure 2.

### 3.4 Effects of TRE on SBP

A total of 11 studies including 353 participants in the control group and 366 in the TRE group were included in the meta-analysis of SBP. The pooled result showed that TRE without CR significantly reduced SBP (WMD = −1.79 mmHg, 95% CI: −3.30 to −0.27, *p* = 0.02). Heterogeneity was negligible (*I*^2^ = 0%, Chi^2^ = 5.65, df = 10, *p* = 0.84; Tau^2^ = 0.00), indicating consistent findings across studies.

**Figure 3:**
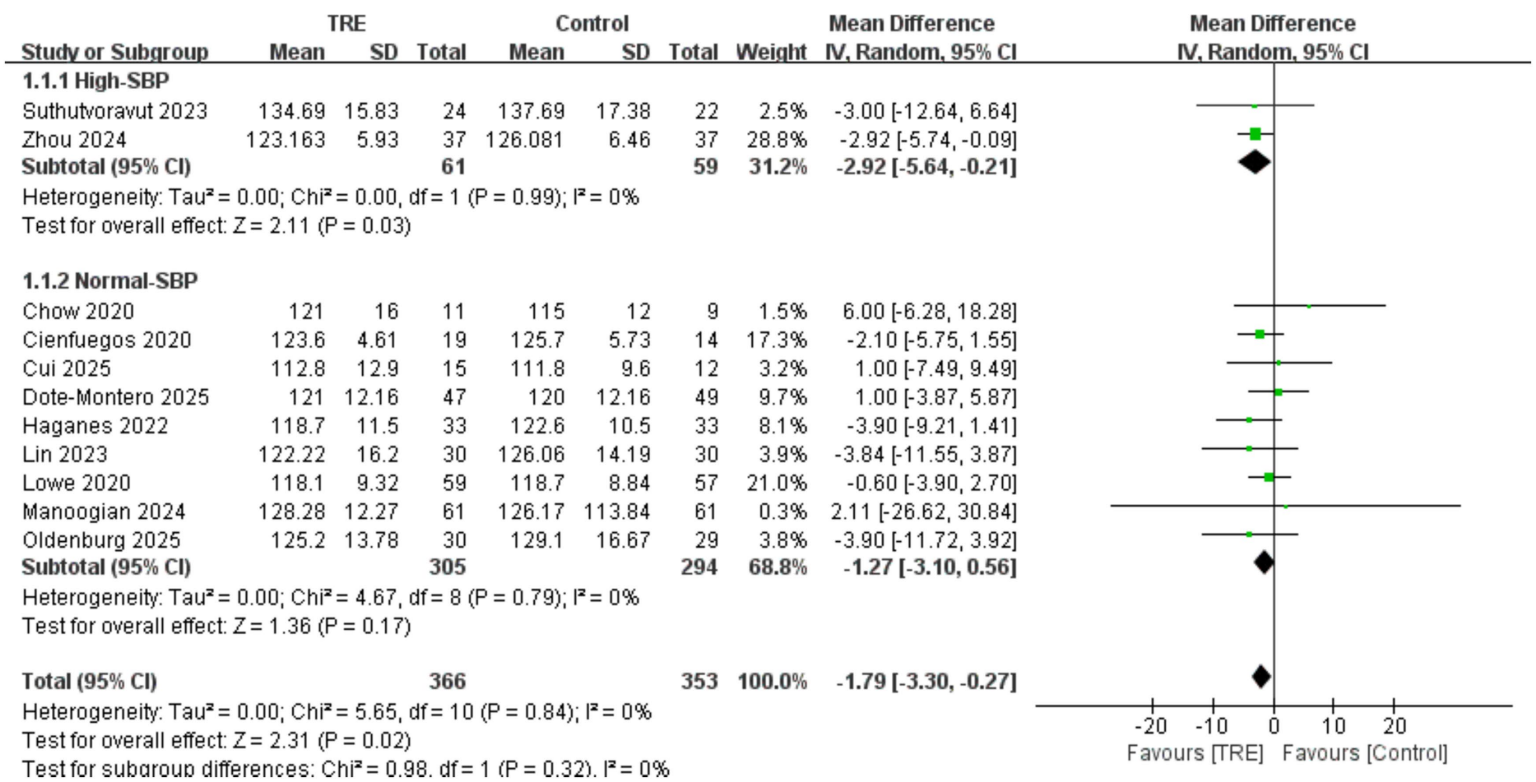
Meta-analysis of the effects of TRE on SBP.

A predefined subgroup analysis was performed based on baseline SBP. Studies were categorized as “high SBP” if both groups had baseline mean SBP ≥ 130 mmHg. In the high-SBP subgroup (2 studies; *n* = 59 control, 61 TRE), the pooled effect was statistically significant: WMD = −2.92 mmHg (95% CI: −5.64 to −0.21, *p* = 0.03), *I*^2^ = 0%, Chi^2^ = 0.00, df = 1, *p* = 0.99; Tau^2^ = 0.00. In the normal-SBP subgroup (9 studies; *n* = 294 control, 305 TRE), the effect was not statistically significant: WMD = −1.27 mmHg (95% CI: −3.10 to 0.56, *p* = 0.17), *I*^2^ = 0%, Chi^2^ = 4.67, df = 8, *p* = 0.79; Tau^2^ = 0.00. While the statistical test for subgroup differences was not significant (Chi^2^ = 0.98, df = 1, *p* = 0.32), it is noteworthy that the BP-lowering effect of TRE was only statistically significant in the high-SBP subgroup (*p* = 0.03), and not in the normal-SBP subgroup (*p* = 0.17). This suggests that TRE may exert greater effects in individuals with elevated baseline SBP. As shown in Figure 3.

### 3.5 Effects of TRE on DBP

Similar to SBP, a total of 11 studies including 353 participants in the control group and 366 in the TRE group were included in the meta-analysis of DBP. The pooled result showed that TRE without CR significantly reduced DBP (WMD = −1.75 mmHg, 95% CI: −3.07 to −0.43, *p* = 0.01). Heterogeneity was low (*I*^2^ = 14%, Chi^2^ = 11.65, df = 10, *p* = 0.31; Tau^2^ = 0.70), indicating mild between-study variability.

**Figure 4:**
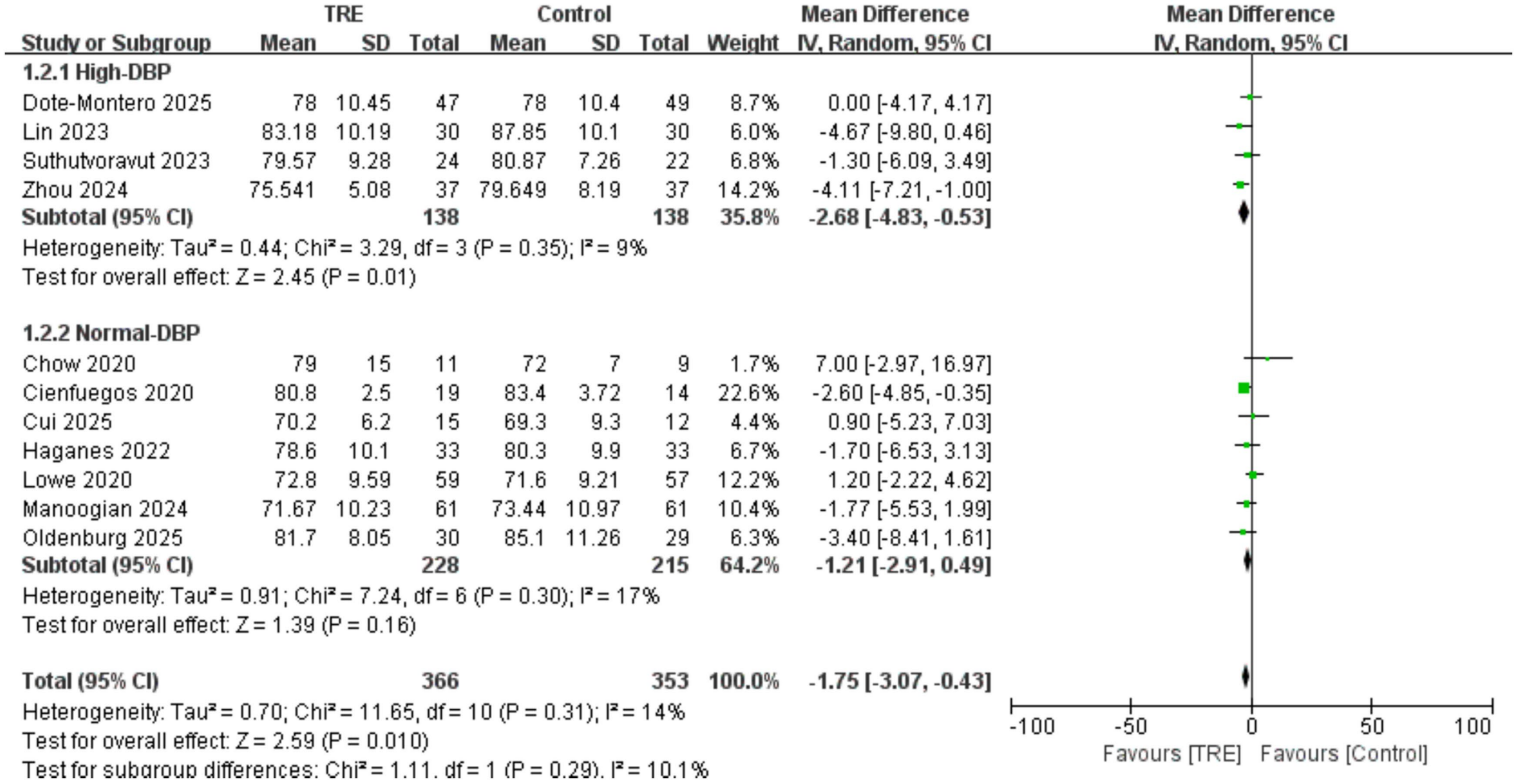
Meta-analysis of the effects of TRE on DBP.

A predefined subgroup analysis was conducted based on baseline DBP. Studies were categorized as “high DBP” if both groups had a mean baseline DBP ≥ 80 mmHg. In the high-DBP subgroup (4 studies; *n* = 138 control, 138 TRE), the pooled effect was statistically significant: WMD = −2.68 mmHg (95% CI: −4.83 to −0.53, *p* = 0.01), *I*^2^ = 9%, Chi^2^ = 3.29, df = 3, *p* = 0.35; Tau^2^ = 0.44. In the normal-DBP subgroup (7 studies; *n* = 215 control, 228 TRE), the effect was not statistically significant: WMD = −1.21 mmHg (95% CI: −2.91 to 0.49, *p* = 0.16), *I*^2^ = 17%, Chi^2^ = 7.24, df = 6, *p* = 0.30; Tau^2^ = 0.91. Although the test for subgroup differences was not significant (Chi^2^ = 1.11, df = 1, *p* = 0.29), the BP-lowering effect of TRE was only significant in the high-DBP subgroup (*p* = 0.01), suggesting that individuals with elevated baseline DBP may benefit more from TRE. As shown in Figure 4.

### 3.6 Effects of TRE on HR

**Figure 5:**
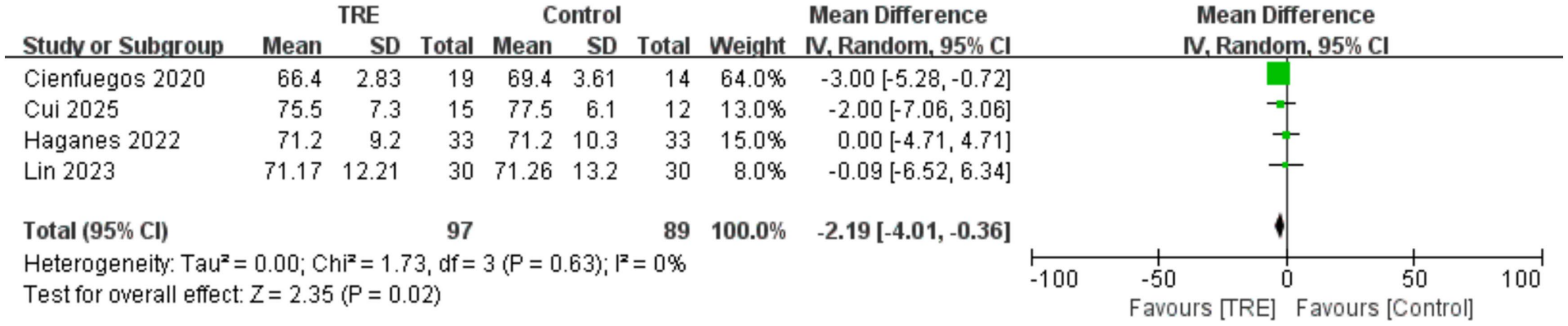
Meta-analysis of the effects of TRE on HR.

Four studies including 89 participants in the control group and 97 in the TRE group were included in the meta-analysis of HR. The pooled analysis showed a statistically significant reduction in HR following TRE without CR (WMD = −2.19 bpm, 95% CI: −4.01 to −0.36, *p* = 0.02). Heterogeneity was negligible (*I*^2^ = 0%, Chi^2^ = 1.73, df = 3, *p* = 0.63; Tau^2^ = 0.00), suggesting consistent results across studies. As shown in Figure 5.

### 3.7 Effects of TRE on FBG

**Figure 6:**
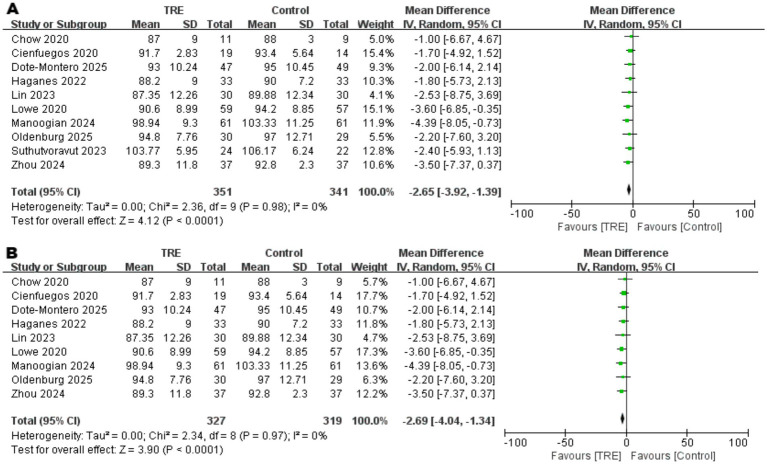
Meta-analysis of the effects of TRE on FBG. Panel (A) shows the main analysis, and Panel **(B)** shows the sensitivity analysis..

Ten studies including 341 participants in the control group and 351 in the TRE group were included in the meta-analysis of fasting blood glucose (FBG). The pooled analysis showed a statistically significant reduction in FBG following TRE without CR (WMD = −2.65 mg/dL, 95% CI: −3.92 to −1.39, *p* < 0.0001). Heterogeneity was negligible (*I*^2^ = 0%, Chi^2^ = 2.36, df = 9, *p* = 0.98; Tau^2^ = 0.00), indicating consistent effects across studies. As shown in Figure 6A.

Although between-study heterogeneity was low, a sensitivity analysis was conducted to account for one study (22) that enrolled participants with impaired fasting glucose (IFG). After excluding this study, the result remained robust and statistically significant (WMD = −2.69 mg/dL, 95% CI: −4.04 to −1.34, *p* < 0.0001; *I*^2^ = 0%), supporting the stability of the observed effect. As shown in Figure 6B.

### 3.8 Effects of TRE on FINS

**Figure 7:**
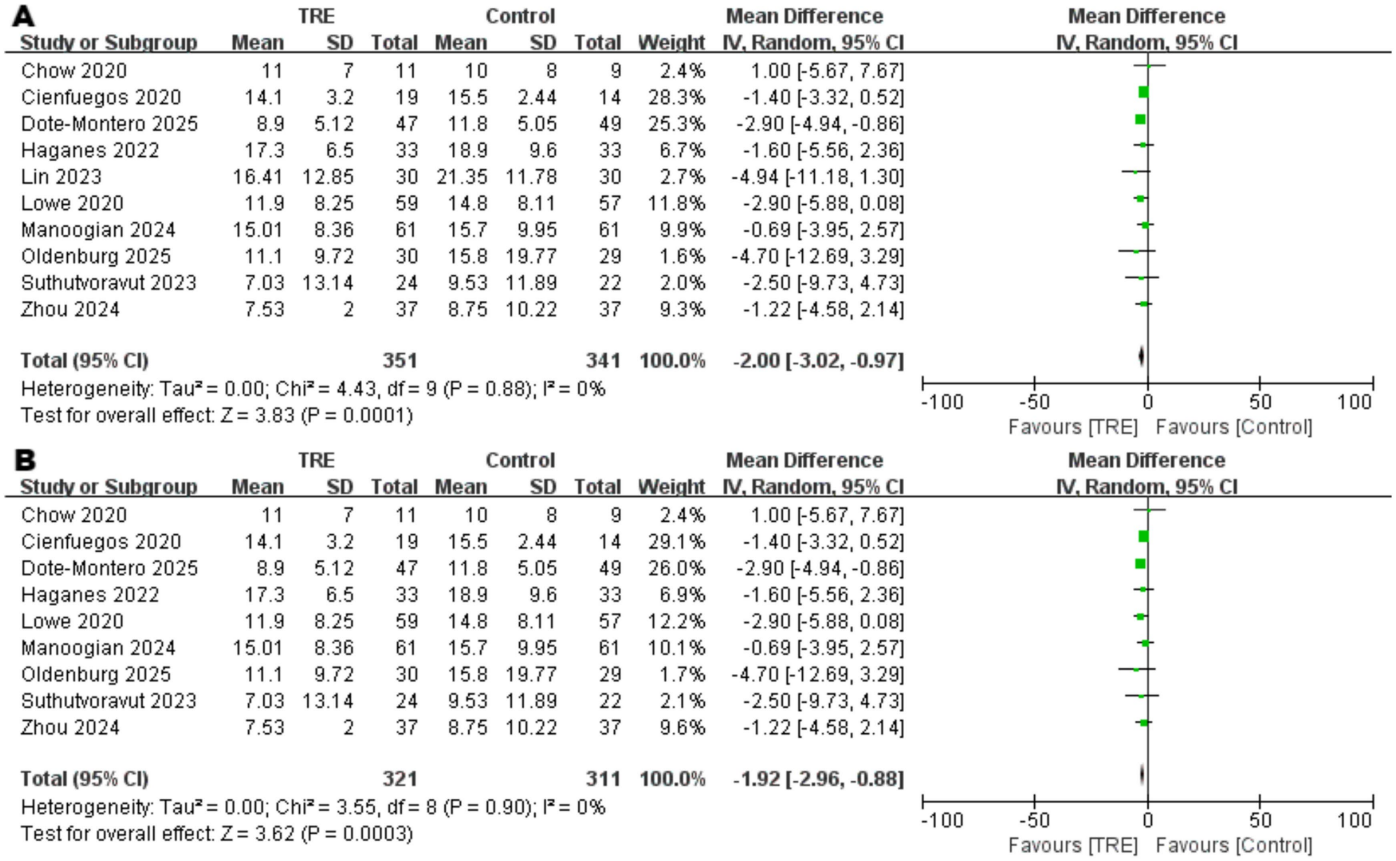
Meta-analysis of the effects of TRE on FINS. Panel **(A)** shows the main analysis, and Panel **(B)** shows the sensitivity analysis.

Ten studies including 341 participants in the control group and 351 in the TRE group were included in the meta-analysis of fasting insulin (FINS). The pooled analysis showed a statistically significant reduction in FINS following TRE without CR (WMD = −2.00 μIU/mL, 95% CI: −3.02 to −0.97, *p* = 0.0001). Heterogeneity was negligible (*I*^2^ = 0%, Chi^2^ = 4.43, df = 9, *p* = 0.88; Tau^2^ = 0.00), indicating consistent findings across studies. As shown in Figure 7A.

Although heterogeneity was low, a sensitivity analysis was conducted by excluding one study (26) that included participants with markedly elevated baseline insulin levels. The result remained statistically significant (WMD = −1.92 μIU/mL, 95% CI: −2.96 to −0.88, *p* = 0.0003; *I*^2^ = 0%), supporting the robustness of the observed effect. As shown in Figure 7B.

### 3.9 Effects of TRE on HOMA-IR

Ten studies including 341 participants in the control group and 351 in the TRE group were included in the meta-analysis of HOMA-IR. The pooled analysis showed a significant reduction in HOMA-IR following TRE without CR (WMD = −0.58, 95% CI: −0.81 to −0.35, *p* < 0.00001).

**Figure 8:**
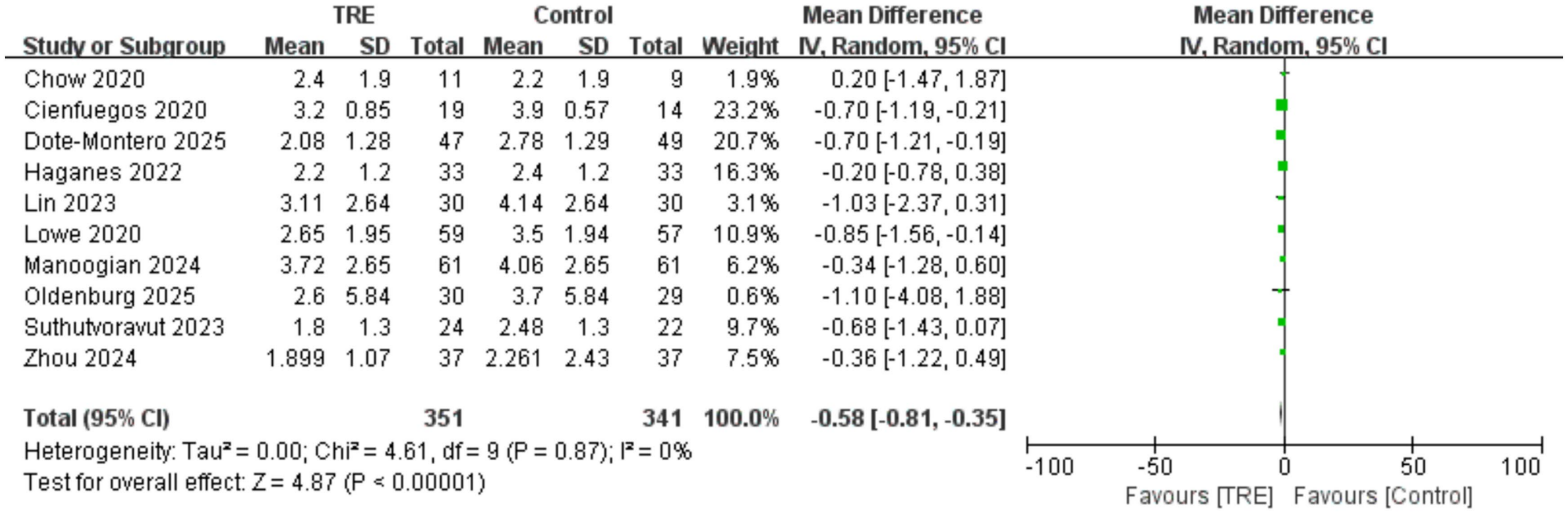
Meta-analysis of the effects of TRE on HOMA-IR.

Heterogeneity was negligible (*I*^2^ = 0%, Chi^2^ = 4.61, df = 9, *p* = 0.87; Tau^2^ = 0.00), indicating highly consistent findings across studies. Subgroup analysis was not conducted, as almost all included studies reported baseline HOMA-IR values ≥2.5 in either the intervention or control group, suggesting that participants were predominantly insulin resistant. As shown in Figure 8.

### 3.10 Effects of TRE on BMI

**Figure 9:**
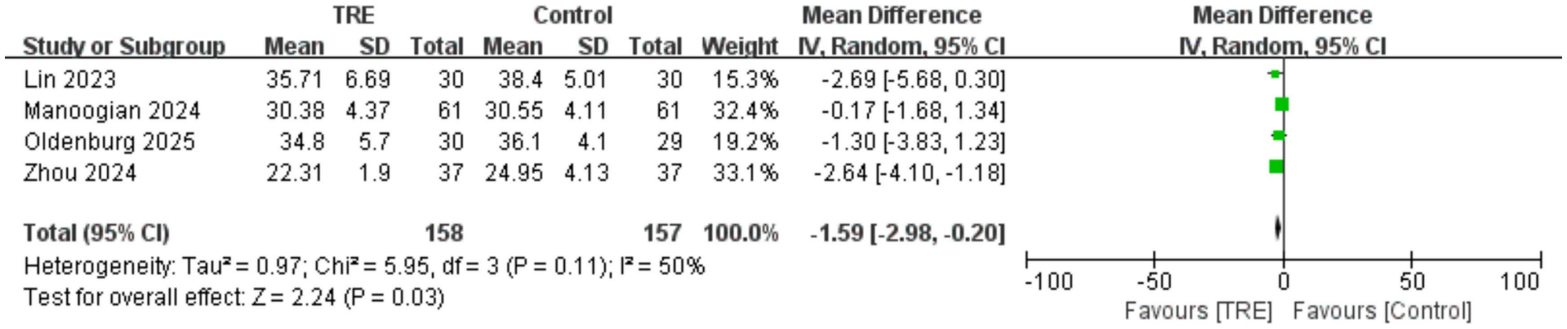
Meta-analysis of the effects of TRE on BMI.

Four studies including 157 participants in the control group and 158 in the TRE group were included in the meta-analysis of BMI. The pooled analysis showed a statistically significant reduction in BMI following TRE without CR (WMD = −1.59 kg/m^2^, 95% CI: −2.98 to −0.20, *p* = 0.03). Heterogeneity was moderate (*I*^2^ = 50%, Chi^2^ = 5.95, df = 3, *p* = 0.11; Tau^2^ = 0.97), indicating some variability in effect sizes across studies. As shown in Figure 9.

### 3.11 Effects of TRE on TC

**Figure 10:**
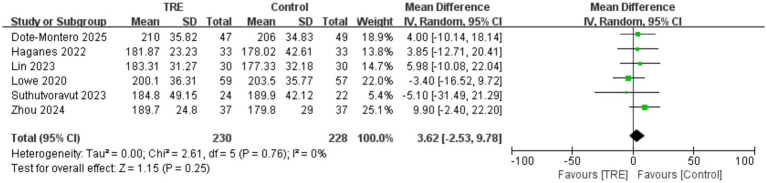
Meta-analysis of the effects of TRE on TC.

Five studies including 228 participants in the control group and 230 in the TRE group were included in the meta-analysis of total cholesterol. The pooled result indicated no statistically significant difference between TRE and control groups (WMD = 3.62 mg/dL, 95% CI: −2.53 to 9.78, *p* = 0.25). Heterogeneity was negligible (*I*^2^ = 0%, Chi^2^ = 2.61, df = 5, *p* = 0.76; Tau^2^ = 0.00), indicating high consistency across included studies. As shown in Figure 10.

### 3.12 Effects of TRE on TG

Ten studies including 341 participants in the control group and 351 in the TRE group were included in the meta-analysis of triglycerides. The pooled analysis showed no statistically significant difference between TRE and control groups (WMD = −4.28 mg/dL, 95% CI: −15.34 to 6.78, *p* = 0.45). Heterogeneity was moderate (*I*^2^ = 51%, Chi^2^ = 18.24, df = 9, *p* = 0.03; Tau^2^ = 133.41), indicating inconsistency across studies.

**Figure 11:**
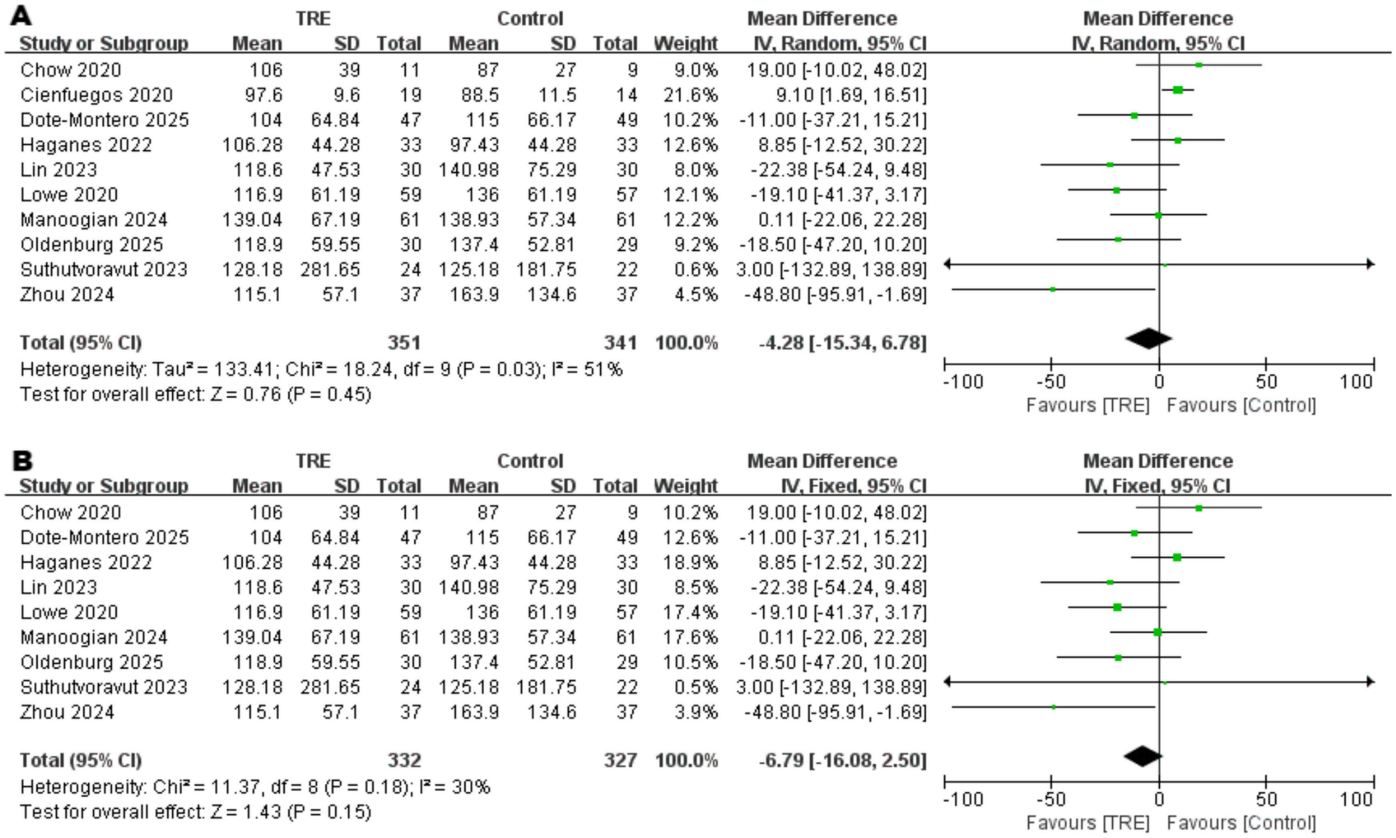
Meta-analysis of the effects of TRE on TG. Panel **(A)** shows the main analysis, and Panel **(B**) shows the sensitivity analysis.

To identify the source of heterogeneity, a leave-one-out sensitivity analysis was performed. When one study (26) was excluded, heterogeneity decreased notably (*I*^2^ = 30%, Chi^2^ = 11.37, df = 8, *p* = 0.18), while the pooled effect remained non-significant (WMD = −6.79 mg/dL, 95% CI: −16.08 to 2.50, *p* = 0.15). As shown in Figure 11A. Closer examination revealed that this study reported unusually small standard deviations for TG (SD = 11.5 in control, 9.6 in TRE), which may have led to disproportionate weighting in the pooled analysis. This pattern suggests that the study may have contributed disproportionately to the pooled estimate and was a likely source of model instability. As shown in Figure 11B.

### 3.13 Effects of TRE on HDL-C

**Figure 12:**
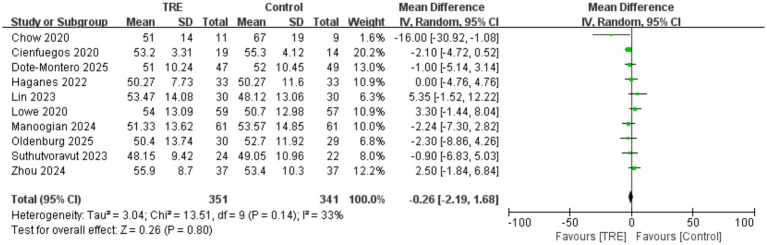
Meta-analysis of the effects of TRE on HDL-C.

Ten studies including 341 participants in the control group and 351 in the TRE group were included in the meta-analysis of HDL cholesterol. The pooled result showed no significant effect of TRE on HDL-C (WMD = −0.26 mg/dL, 95% CI: −2.19 to 1.68, *p* = 0.80). Heterogeneity was low to moderate (*I*^2^ = 33%, Chi^2^ = 13.51, df = 9, *p* = 0.14; Tau^2^ = 3.04), indicating acceptable consistency across studies. As shown in Figure 12.

### 3.14 Effects of TRE on LDL-C

**Figure 13:**
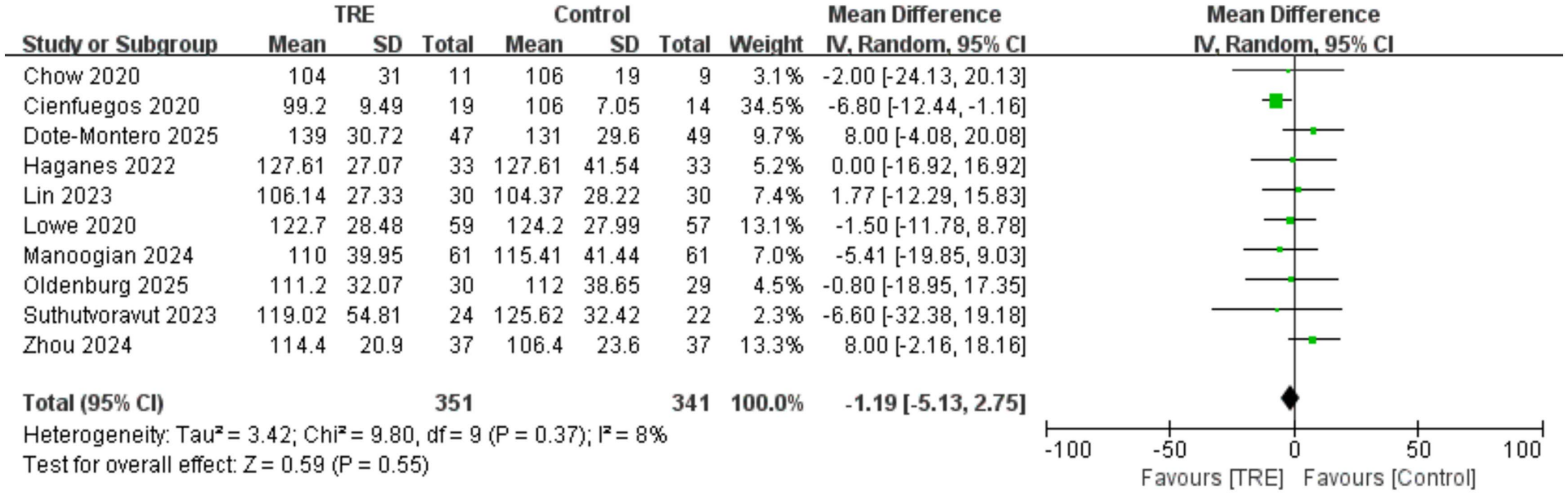
Meta-analysis of the effects of TRE on LDL-C.

Ten studies including 341 participants in the control group and 351 in the TRE group were included in the meta-analysis of LDL cholesterol. The pooled analysis showed no significant change in LDL-C following TRE (WMD = −1.19 mg/dL, 95% CI: −5.13 to 2.75, *p* = 0.55). Heterogeneity was very low (I^2^ = 8%, Chi^2^ = 9.80, df = 9, *p* = 0.37; Tau^2^ = 3.42), suggesting consistent findings. As shown in Figure 13.

### 3.15 Funnel plots

**Figure 14:**
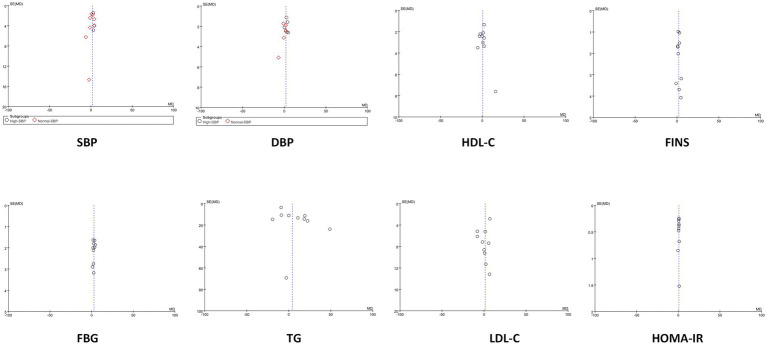
Funnel plots.

For outcomes with at least 10 included studies, funnel plots were used to assess potential publication bias. The funnel plots for all outcomes appeared symmetric or approximately symmetric, indicating a low likelihood of publication bias. This suggests that the studies included in the analysis are less likely to have been selectively published based on their results, and that the overall findings are likely to be unbiased. As shown in Figure 14.

## 4 Discussion

The association between BP levels and the development and progression of various CVDs is well established (30). Our meta-analysis found that TRE without CR effectively reduced both SBP and DBP in non-diabetic adults. However, subgroup analyses revealed that while TRE significantly lowered SBP and DBP in hypertensive individuals, it did not exert a significant effect on normotensive individuals. These findings suggest that the observed reductions in SBP and DBP may be primarily driven by participants with elevated baseline BP, indicating a potentially greater responsiveness to TRE in hypertensive populations. This result is consistent with our previous animal study (31), which demonstrated that 16 weeks of time-restricted feeding (TRF), with a feeding window from 9:00 a.m. to 5:00 p.m., significantly reduced BP in spontaneously hypertensive rats (SHRs), but had no marked effect on normotensive Wistar-Kyoto (WKY) rats. These findings imply that the antihypertensive effect of TRE may be related to the body’s homeostatic state. Under hypertensive conditions, where homeostasis is disrupted, TRE may correct this imbalance through multiple mechanisms, leading to BP reduction. In contrast, in individuals with stable physiological conditions, TRE does not excessively modulate BP, thereby avoiding potential adverse effects from unnecessary BP reduction. Several prior meta-analysis (32, 33) have shown that CR can lower both SBP and DBP. In contrast, our study suggests that even without CR, TRE may still confer benefits in lowering BP, particularly in hypertensive populations. Unlike CR, which often leads to significant feelings of hunger and reduced patient adherence, TRE offers a more feasible and sustainable approach. This makes it a promising non-pharmacological strategy for managing hypertension, especially in patients who may struggle with the subjective discomfort of CR. However, the underlying mechanisms of TRE’s antihypertensive effects remain incompletely understood. Although some animal studies suggest that TRF may exert its effects by suppressing the renin-angiotensin system (RAS), inhibiting inflammation or regulating autophagy (31, 34, 35), large-scale clinical trials are needed to confirm these findings. Elucidating the mechanisms of TRE-induced BP reduction will be important for its clinical translation and for evaluating its safety profile. Additionally, our analysis also observed that TRE may lead to a reduction in HR, which consistent with the previous clinical study (14) and aligns with outcomes reported in studies investigating CR intervention (32). Nonetheless, due to the limited number of included studies reporting HR outcomes, further research is warranted to confirm the effect of TRE on HR.

Disorders of glucose metabolism and insulin resistance are also recognized as significant risk factors for CVDs (36, 37). Our study demonstrated that TRE without CR significantly reduced FBG, FINS, HbA1c, and HOMA-IR. Notably, subgroup analysis revealed that the reductions in FBG and FINS remained statistically significant even after excluding different studies with elevated baseline levels - one study (22) with a high baseline FBG and another (26) with a markedly high baseline FINS. This suggests a more pronounced suppressive effect of TRE on FBG and FINS, while also indicating a potential risk of hypoglycemia in individuals with normal glucose levels. Therefore, when implementing TRE in clinical practice, caution should be exercised to avoid excessive reductions in blood glucose that may lead to adverse outcomes. A recent meta-analysis evaluating TRE with CR (38) suggests that combining TRE with CR effectively lowers FBG levels and improves insulin resistance. Our findings show that TRE alone, without CR, can achieve similar metabolic improvements, highlighting the potential of TRE as an independent strategy for enhancing metabolic health. Taken together, these effects may contribute to the cardiovascular benefits associated with TRE, supporting its potential as an effective intervention for reducing CVD risk.

Dyslipidemia, mainly characterized by elevated TC, TG, and LDL-C levels and reduced HDL-C, is an independent risk factor for CVDs (39, 40). In our study, calorie-unrestricted TRE did not show significant effects on lipid profiles, which contrasts with findings from CR interventions. Several meta-analysis (41–43) have shown that CR is effective in improving lipid profiles, likely due to its overall energy-reducing effects. The absence of such changes with TRE in our study may be attributed to the fact that TRE, by itself, only limits the eating window without reducing overall caloric intake. This suggests that while TRE may help improve certain metabolic parameters, such as blood glucose control, it might not exert sufficient effects on lipid metabolism without concurrent caloric restriction. Therefore, if the goal is to improve lipid profiles, relying on calorie-unrestricted TRE alone may not be sufficient. In addition, other forms of intermittent fasting, such as alternate day fasting, have been shown to improve lipid profiles (44), possibly due to the longer fasting duration compared to TRE. This highlights potential differences in efficacy among various intermittent fasting regimens. Moreover, our study found that TRE was associated with reductions in BMI; however, due to the limited number of included studies and the relatively high baseline BMI, further research is needed to confirm this finding. In conclusion, while calorie-unrestricted TRE may reduce BMI, it does not significantly improve lipid profiles and therefore may not enhance cardiac health through improvements in lipid metabolism. An important consideration emerging from our analysis is the consistent observation of modest weight loss, as indicated by reduced BMI, despite the theoretically isocaloric design of the included studies. This suggests that in practice, strict energy balance was not maintained, likely due to spontaneous reductions in energy intake under the TRE regimen (45). Consequently, the observed weight loss must be recognized as a potential confounder when attributing cardiometabolic effects solely to meal timing. This may partially explain the divergent results across outcomes: significant improvements in BP and glucose metabolism, contrasted with the null effects on lipid profiles. The current data cannot differentiate whether weight loss preferentially affects muscle mass, subcutaneous fat, or visceral fat, which limits interpretation of body composition changes under TRE. Lipid metabolism typically requires more substantial energy deficit or different dietary composition for meaningful modification (23). Furthermore, the applicability of our findings is specifically relevant to individuals with overweight or obesity, for whom such unintentional weight loss is likely advantageous. The effects of TRE on normal-weight populations remain uncertain and warrant dedicated investigation, as unintended weight loss in this group could potentially be harmful.

From a clinical perspective, the findings of this meta-analysis carry important implications for cardiovascular risk management and lifestyle-based prevention strategies. The observed reductions in BP and improvements in glucose metabolism indicate that TRE could serve as a practical, low-cost, and non-pharmacological intervention for individuals at risk of hypertension, metabolic syndrome, or prediabetes. Compared with traditional CR, which is often associated with poor adherence and subjective discomfort, TRE provides a more sustainable and patient-friendly approach that can be feasibly integrated into daily routines. Moreover, the greater responsiveness observed in hypertensive and insulin-resistant individuals suggests that TRE may offer targeted benefits for populations with metabolic dysregulation, underscoring the importance of personalized dietary recommendations. Incorporating TRE into comprehensive lifestyle modification programs could therefore enhance cardiovascular prevention efforts and potentially reduce the long-term burden of cardiometabolic diseases in clinical settings.

This meta-analysis has several limitations that warrant consideration. First, the number of included studies was relatively small, particularly for certain outcomes such as HR and BMI, which may limit the statistical power and generalizability of our findings. Second, significant heterogeneity must be emphasized, particularly in the eating window durations, its daily timing, and the meal frequency, which was not reported in any studies. These differences preclude definitive conclusions regarding the influence of any single TRE parameter and highlight the need for future studies to systematically compare these factors. Third, the lack of blinding and potential reporting bias in some included trials could have affected the validity of the results. Despite these limitations, our study has several strengths. Fourth, the observed weight loss, although modest, indicates that the TRE intervention were not perfectly isocaloric in practice. While this likely contributed to the observed benefits on BP and FBG, it also introduces a potential confounder, and the precise contribution of meal timing independent of energy deficit remains to be fully elucidated. To our knowledge, this is the first meta-analysis to systematically evaluate the impact of calorie-unrestricted TRE on comprehensive cardiometabolic outcomes in non-diabetic adults. By performing subgroup analyses based on baseline health status, we were able to provide novel insights into the population-specific effects of TRE, particularly highlighting its potential benefits in individuals with elevated BP or impaired glucose metabolism. Furthermore, we critically compared our findings with both clinical and preclinical data, offering a more nuanced understanding of the physiological responses to TRE.

## 5 Conclusion

In conclusion, our meta-analysis demonstrates that TRE without CR can significantly reduce BP and improve glucose metabolism in non-diabetic adults, particularly in those with pre-existing high BP or high FBG or high FINS. However, TRE does not appear to exert meaningful effects on lipid profiles in the absence of CR, indicating that its cardiometabolic benefits may be selective rather than comprehensive. These findings underscore the potential of TRE as a non-pharmacological intervention for the management of hypertension and glucose homeostasis, but also highlight the need for personalized approaches and careful monitoring, especially in normotensive or normoglycemic individuals. Future large-scale, long-term randomized controlled trials are essential to validate these findings, elucidate underlying mechanisms, and determine the optimal TRE protocols for different populations.

## Data Availability

The original contributions presented in the study are included in the article/supplementary material, further inquiries can be directed to the corresponding author.
